# Effect of Membrane Fouling on Fertilizer-Drawn Forward Osmosis Desalination Performance

**DOI:** 10.3390/membranes10090243

**Published:** 2020-09-18

**Authors:** Majeda Khraisheh, Mona Gulied, Fares AlMomani

**Affiliations:** Department of Chemical Engineering, College of Engineering, Qatar University, Doha P.O. Box 2713, Qatar; mgulied@qu.edu.qa (M.G.); falmomani@qu.edu.qa (F.A.)

**Keywords:** forward osmosis, fertilizer draw solute, fouling, aquaculture wastewater, desalination

## Abstract

Fertilizer-drawn forward osmosis (FDFO) has garnered immense attention for its application in the agricultural field and its potential to reuse wastewater sustainably. Membrane fouling, however, remains to be a challenge for the process. This study aims to investigate the influence of membrane fouling on the performance of the FDFO process. Synthetic wastewater (SWW) and multi-component fertilizer (MCF) were used as feed solution (FS) and draw solution (DS) with cellulose triacetate (CTA) forward osmosis (FO) membrane orientation. The performance was evaluated through water flux (WF), percentage recovery and percentage of salt reject. The WF declined from 10.32 LMH (L/m^2^·h) to 3.30 LMH when ultra-pure water as FS was switched with concentration FS indicating the dependence of the performance on the type of FS used. Accelerated fouling experiments conducted to verify the fouling behavior showed a decline in the water flux from 8.6 LMH to 3.09 LMH with SWW and 13.1 LMH to 3.42 LMH when deionized water was used as FS. The effects of osmotic backwashing and in situ flushing as physical cleaning methods of the foul membrane were studied through water flux and salt recovery percentage. Both cleaning methods yielded a WF close to the baseline. Osmotic backwashing yielded better results by eliminating foulant–foulant and foulant–membrane adhesion. The cleaning methods were able to recover 75% of phosphate and 60% of nitrate salts. Scanning electron microscopy (SEM), atomic force microscopy (AFM) and Fourier transform infrared (FTIR) results validated the effectiveness of the methods for the physical cleaning of foul membranes. This study underlines the importance of the FS used in FDFO and the effectiveness of osmotic backwashing as a cleaning method of FO membranes.

## 1. Introduction

In times of ever-increasing water scarcity, membrane-based desalination has been sought among the most efficient desalination processes available. Reverse osmosis (RO) and nano-filtration (NF), in particular, have been widely used across the globe for the desalination of seawater and brackish water [1,2]. However, a major concern is the inevitable fouling of membranes used in the process. The scaling of membranes not only shortens the life of the porous material; it also hinders efficient operation, consequently leading to increased energy consumption [3,4]. Furthermore, the scaling of membranes is linked to increase in operation and maintenance cost as well [5,6].

Forward osmosis (FO) has garnered immense attention for its application in water desalination, especially as an energy efficient and economical alternative to conventional membrane-based desalination methods. Defined as the state-of-the-art process [3], FO employs the natural osmotic pressure as the driving force. The absence of hydraulic pressure in the process indicates that the FO technology uses osmotic differential for separation across the membrane, unlike RO and NF processes. The osmotic differential causes freshwater molecules to flow from the feed solution to the draw solution through the semi permeable membrane, resulting in concentrated Feed solution and diluted draw solution. Perhaps the most attractive feature of the process is the low fouling propensity of the FO membranes and its ease of cleaning. Although fouling is inescapable, several studies have claimed, both organic and inorganic membrane fouling in FO are entirely reversible with use of physical cleaning [7,8,9,10,11,12,13,14,15,16]. Though the fouling propensity of the FO process is lower than that of other pressure-driven membrane processes, studies have been conducted to understand the FO fouling mechanism, the factors influencing FO membrane fouling and methods to mitigate them [4,15,16,17,18]. 

The FO fouling mechanism was compared to that of RO for better comprehension [2]. Lee et al. [7] studied the fouling behavior of FO and RO, to conclude that the key fouling mechanism in FO is the accelerated cake-enhanced osmotic pressure (CEOP). Siddiqui et al. [19] claim that FO and RO, due to the difference in their driving force, behave differently to the progression of fouling. It was concluded that the foulant resistance in FO was greater than that of RO due to the change in internal concentration polarization (ICP) and the driving force to increasing fouling. Emphasis therefore is paid to selecting the appropriate draw solution (DS) and membrane properties for efficient FO performance. 

Wang et al. [20] found that increasing the DS concentration could aggravate membrane fouling. However, it was discussed that the crossflow velocity plays a greater role in membrane fouling than DS concentration. The effect of DS concentration on water flux was examined by Zou et al. [21]. Increased DS concentration leads to an increase in water flux. High water flux is then linked to high permeation drag force and therefore increased foulant concentration near the surface of the membrane. Furthermore, the increased water flux accelerates feed solution (FS) concentration and DS dilution leading to a heavy decline in initial flux [12,17].

Siddiqui et al. [19] upon comparing the fouling mechanisms of RO and FO, claimed fouling in FO is dependent on the foulants present in the FS. This claim was verified against the findings of Zou et al. [21] that the presence of certain ions such as calcium and magnesium may exacerbate membrane fouling through the formation of complexes with other functional groups. 

Following the fouling mechanism of FO processes, several studies have been dedicated to study the mitigation of fouling in FO processes. The common observation among these studies was the reversibility of FO membrane fouling. Phuntsho et al. [5] found that the absence of hydraulic pressure in FO process causes organic foulants to loosely accumulate on the membrane. This thick and sparse foul layer can be eliminated through simple physical cleaning methods such as hydraulic flushing. They claim that high crossflow velocity was linked to greater shear stress near the membrane surface. This consequently reduces the foulants formed on the membrane. Moreover, the reduced external concentration polarization (ECP) with increased crossflow velocity improves membrane cleaning efficiency. Studies conducted by Lotfi et al. [13] yielded verifiable results. Kim et al. [17], presented that high crossflow velocity generated low concentration polarization, causing an increase in water flux. This consequently is associated with a high-permeation drag force and may lead to more thick and dense fouling near the FO membrane. They suggested employing osmotic backwashing as an alternative for membrane cleaning. It has also been reported that osmotic backwashing can be effectively used for colloidal fouling as well as combined organic and colloidal fouling removal when cellulose triacetate (CTA) FO membrane is used. 

Currently, FO technology has been increasingly studied and applied for agricultural purposes. The draw solution consisting of a mix of diluted fertilizer and desalinated water mix eliminates the need for scarce fresh water for irrigation while also desalinating seawater or brackish water. The key parameter of the success of the FO process for use in agriculture is the choice of draw solution as can be verified by the aforementioned literature. The effects of the DS on the performance of the fertilizer-drawn forward osmosis (FDFO) studied by Chekli et al. [22] using single fertilizers, while Phuntsho et al. [6] examined the same using a blend of fertilizers. It was concluded that the choice of fertilizers in the DS affects the concentration polarization (CP) and consequently the water flux. Several studies reported that most commercially available fertilizers generate the required high osmotic pressure required for FO and can thus be used as a suitable draw solution.

To date, a few studies on the FDFO system demonstrated a comparison between different physical cleaning approaches to evaluate the system performance, whereas most studies on the FDFO system have employed one of the physical cleaning approaches depending on the type or source of FS. These studies reported that the hydraulic in situ flushing is suitable for the FDFO system operated with FS that has low salinity and negligible organic contaminates [23]. Accordingly, this study examined the effect of membrane fouling by using a CTA membrane oriented in FO mode (the active layer facing the FS) on the performance of the FDFO system. The performance was primarily investigated through its water flux, water recovery percentage and salt flux percentage. Marine aquaculture wastewater and multi-component fertilizer salts were used as FS and DS, respectively. Furthermore, the effectiveness of osmotic backwashing and in situ flushing were also analyzed. 

## 2. Material and Methods 

### 2.1. Chemicals

The chemicals used in the experiment were sodium chloride (NaCl, 99%), magnesium chloride (MgCl_2_, 99%) and calcium chloride (CaCl_2_, 99%). These chemicals were of analytical grade (ACS grade, ISO 17025, Sigma Aldrich, Doha, Qatar) and used without modifications.

### 2.2. Feed and Draw Solution

Synthetic wastewater (SWW) and multi-components fertilizer (MCF) salt prepared from commercial fertilizer salt (Arab Qatari Agriculture Company) were utilized as feed solution and draw solute to the FO system, respectively. The SWW was prepared to mimic marine aquaculture effluent by dissolving 100,000 mg of commercial fertilizer salt (NPK), 116,000 mg of NaCl, 2000 mg of MgCl_2_ and 2000 mg of CaCl_2_ in 4 L of distilled water (DW). The water characteristic for SWW is presented in Table 1. The multi-component fertilizer draw solution (MCFDS) was prepared by dissolving 150,000 mg of commercial fertilizer salt (NPK) in 5 L of DW, which corresponds to 135.62 bar of osmotic pressure. The composition of commercial fertilizer salt is presented in Table 2. The osmatic pressure (π) of the DS and FS was obtained by Aspen plus (Aspen One^®^ Version 9 Software Aspen technology, Inc., Bedford, MA, USA). 

### 2.3. Forward Osmosis Membrane

This study was conducted using a commercial flat sheet CTA FO membrane with a surface area of 0.90 cm^2^ obtained from the Fluid Technology Solution (FTSH2), Albany, OR, USA. The CTA FO membrane has been utilized in several studies of the FDFO system, since it can tolerate a pH level between 3 and 7 pH and a maximum operating temperature of 50 °C.

### 2.4. Experimental Setup

**Figure 1:**
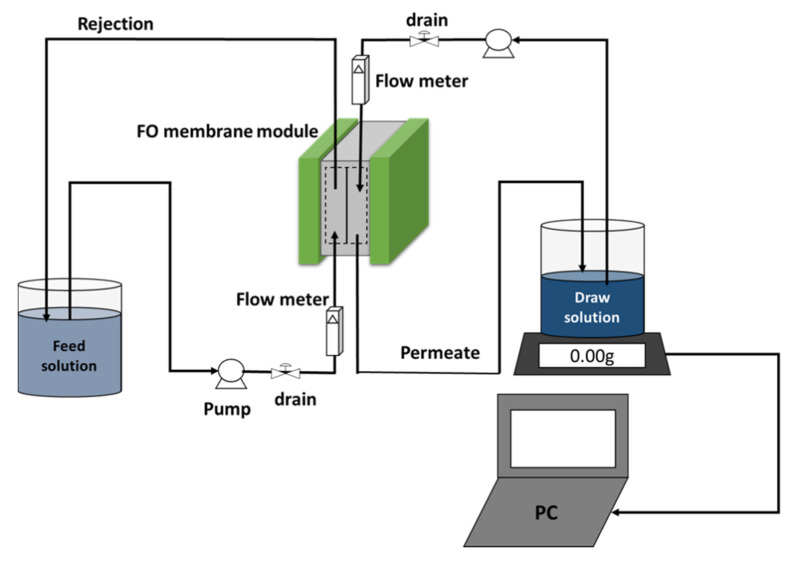
Forward osmosis (FO) experimental setup bench-scale.

The FO bench-scale unit presented in Figure 1 was used in the fouling experiments and consisted of a flat sheet FO membrane cell (Sepa CF cell, Stertlitech Inc., Kent, WA, USA), variable speed gear pumps (Cole-Parmer, Vernon Hills, IL, USA), flow meters (OMECA, Engineering, Kent, WA, USA), 3/8 steel tube fittings (Sterlitech Inc., Kent, WA, USA) feed and draw solution reservoir, pressure gauges with T fittings (Sterlitech Inc., Kent, WA, USA). A more detailed description of the FO experimental unit is provided elsewhere [3].

### 2.5. Control Experiment and Membrane Fouling Test 

The FO membrane experiments were carried out with the membrane oriented in FO-mode (active layer (AL) facing the FS and porous layer (PL) facing the DS). For system stability, deionized (DI) water was used as the FS and DS to be circulated at a constant flowrate for 2 h. All FO runs were conducted at room temperature (25 ± 2 °C) and without the mixing of FS and DS for 24 h, and the circulation flowrate was kept constant at 1.6 L/min (LPM) for both FS and DS. A prior membrane fouling test, as control experiment, was performed by using DI water as FS and MCF as DS, in order to use it as baseline for later comparison. Fouling tests were carried out by using SWW as FS and a highly concentrated DS (300 g/L) was utilized to maximize the water flux and to accelerate the fouling rate.

A new FO membrane was installed in the FO membrane cell before each fouling test, and the system was rinsed with DI water for 30 min. After each fouling test, the FO membrane was cleaned by using physical cleaning for 30 min to recover the permeate flux and water samples were collected from the FS and DS reservoir for analysis. To validate the reliability of the experimental data, each FO run was repeated three times and the average results were considered in calculation at 95% confidence level. 

### 2.6. Physical Cleaning Approach 

Two fouling control techniques were adopted in this study, namely osmotic backwash and in-situ flushing. The osmotic backwash is a physical cleaning process where the FS and DS were replaced by NaCl (1 M) and DI water, respectively. As for in situ, flushing (forward washing) was achieved by using DI water for both FS and DS. The applied experimental conditions to calculate the water flux after cleaning were similar to those used to determine the initial pure water flux for the clean membranes. To evaluate the effectiveness of each physical cleaning approach, an accelerated membrane fouling experiment was first performed and after that, the membrane was cleaned for 30 min once by osmotic backwash or in situ flushing at a fixed circulation flowrate (l.6 LPM). Each fouling control approach was performed separately and continually for membrane cleaning and water flux recovery.

### 2.7. Measurement and Analysis 

The water quality parameters of FS and DS were measured prior and after FO following standard procedures. The electrical conductivity and pH were monitored using OrionTM Versa Star ProTM Benchtop Meter (ThermoFisher Scientific, Waltham, MA, USA) and the key ions’ concentration were determined by using ion chromatography (IC) (850 Professional Mertohm, Switzerland). The Total organic carbon (TOC) was analyzed using Formacs TOC/TN analyzer by Skalar Analyzer (Brtda, The Netherlands). The morphology and structure of the FO membrane surface was characterized using scanning electron microscopy (SEM, NOVA NANO SEM 450, USA), atomic force microscopes (AFM, ASYLUM RESEARCH, UK) and Fourier transform infrared (FTIR) spectroscopy (PerkinElmer Spectrum 400 m USA). The average water flux (*J_w_*) and specific water flux (*J_w_*) were calculated using the volume change of DS per unit area and time as shown in Equations (1) and (2), respectively [24]:

(1) $$J_{w}=\frac{\Delta V}{A_{eff}\cdot \Delta t}$$

(2) $$J_{w}=\frac{\Delta V}{A_{eff}\cdot t}$$

The percentage of water recovered from FS is determined by dividing the volumetric flowrate (*Q_P_*) of the permeate solution over the volumetric flowrate of FS (*Q_F_*), as expressed in Equation (3) [25]:

(3) $$\mathrm{Water}\;\mathrm{recovery}\;\%=\frac{Q_{p}}{Q_{F}}\times 100$$

The water flux recovery % is calculated using Equation (4), where *J_c_* is the initial water flux after cleaning (backwash/in situ flushing) and *J_o_* is the initial water flux of the new membrane used for the first time for a fouling experiment [26]:

(4) $$\%\mathrm{Fluxrecovery}=\frac{J_{c}}{J_{o}}\times 100$$

## 3. Results and Discussion

### 3.1. Membrane Fouling 

**Figure 2:**
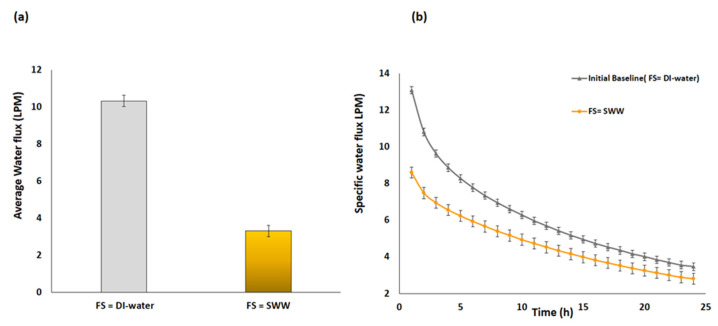
(**a**) Average water flux (**b**) specific water flux. Conditions: feed solution (FS) = SWW, multi-component fertilizer draw solution (MCFDS) (300 g/L) and FS and draw solution (DS) flowrate = 1.6 L/min (LPM) at room temperature for 24 h operating time. Baseline represents the control experiment where FS= deionized (DI) water, MCFDS (300 g/L) at fixed flowrate = 1.6 LPM for 24 h.

The performance of the FDFO was first assessed through the initial water flux (WF) of the CTA FO membrane when the DI water and SWW were used as FS and MCFDS. Figure 2 describes the decline in average WF and specific water flux (SWF) for different type of feed solution (FSs) at fixed operating conditions. MCFDS has a greater osmotic pressure (OP) (135.62 bar) than the DI water and SWW (37.96 bar) and therefore both FSs were found to generate an initial water flux of 13.1 L/m^2^·h (LMH) and 8.6 LMH, respectively. It was observed in Figure 2a that the average WF sharply declined from 10.32 L/m^2^·h (LMH) to 3.30 LMH when ultrapure water and concentrated solution were used as FS, respectively. This behavior indicates that the type of FS selected has a significant impact on the performance of FDFO. Unlike the SWW, DI water has a negligible OP and contains no foulants, therefore, a greater amount of water was recovered from the feed side. On the other hand, SWW contains fouling precursors such as Ca, Mg, and P that likely promoted membrane fouling and gypsum scaling, consequently decreasing the WF. Gypsum scaling is commonly encountered in the desalination of seawater and brackish water. Therefore, the build-up of the crystallization layer deposited on the surface of CTA FO membrane is another reason that simulated the drop of initial WF when SWW was used as FS. A study conducted by Siddiqui et al. [19] yielded verifiable results pertaining the dependence of flux on the FS used. Furthermore, the presence of Ca ions in the FS could promote the formation of a compact, dense and cross-linked foul layer that could hinder flux. Similarly, Yangali-Quintanilla et al. [24] demonstrated an experimental and simulation approach of seawater (SW) FO desalination and revealed that a concentrated FS, such as SW, has a major influence on the permeation of recovered water across the surface. In another study, Xie et al. [27] quantified the gypsum scaling between the commercial FO membrane such as CTA and the thin-film composite (TFC) polyamide membrane. This study revealed that at the same initial WF, the CTA membrane exhibited lower initial WF decline than the TFC membrane, since the gypsum scaling was more severe in the TFC membrane than in the CTA membrane.

Figure 2b presents the water flux as a function of time for the initial FO run followed by another run without cleaning. The specific water flux (SWF) for DI water and SWW was noted to gradually decline from 13.1 LMH and 8.6 LMH until they stabilized at 3.42 LMH and 3.09 LMH, respectively. The gradual decrease in SWF was due to the continuous dilution of recovered water from the feed side to the draw side. Moreover, the continuous dilution over time dilutes the MCFDS to some extent, which consequently affects the actual driven force of the FO process across the membrane and causes a drop in the WF. In addition, the SWW shower lower SWF values in comparison with the DI water. Such a case resulted due to the lower osmotic pressure difference between the MCFDS and SWW than with pure water [27]. Therefore, the MCFDS was able to draw more water from the DI water when used as an FS and hence the higher water flux.

The drop in WF is also attributed to the membrane-based transport phenomena of concentration polarization (CP). The impact of the concentrative ECP (CECP) on the effective osmotic driving force is strongly influenced by the type of FS. The effect of CECP becomes considerable when the SWW was used as FS, which led to a solute buildup near the surface AL of the membrane and thereby decreased the WF. In addition, the reduction of WF mainly attributed the effect of dilutive ICP (DICP). DICP become significant when the PL of the FO membrane becomes diluted, due to the high dilution rate across the membrane, as a result of the high OP difference between DS (135.62 bar) and FS (37.96 bar). Consequently, the effective driving force becomes weak over time and causes a partial flux compensation, as well as foulant accumulation over the membrane surface. 

To minimize the effects of the external CP, crossflow filtration producing sufficient shear should be employed [28]. CP in FO can occur within the porous support layer; this phenomenon is known as internal concentration polarization. It was observed that the enhanced dilution of the DS within the porous layer plays a significant role in the decline of the osmotic pressure difference thereby having a more pronounced effect on flux decline. Ansari et al. [29] investigated the fouling during the simultaneous osmotic dilution of synthetic wastewater as FS for 24 h. It was reported that the WF decline is related to the decrease in driving force and the formation of fouling that resists water permeation. The same finding was reported by Liu et al., where they evaluated the permeate flux of the membrane after 30 days of operation [18]. Furthermore, the greater flux decline in the DI water curve compared to the SWW curve is also attributed to reverse salt diffusion [30]. This is due to the concentration gradient of salinity becoming greater when DI water is used as the FS. 

The decline in flux due to fouling is mainly caused by organic and inorganic foulants. Colloidal foulants have been reported to cause the lowest flux decline [17] The cake-enhanced osmotic pressure (CEOP) causes colloidal fouling to have the lowest flux decline, despite its dense structure. The CEOP, a dominant factor for flux decline, relies on the thickness of the fouling layer. Therefore, it can be concluded that the flux decline, due to colloidal fouling, is significantly affected by the size of colloids present in the FDFO process. 

### 3.2. Effect of Physical Cleaning on Foul Membrane

#### 3.2.1. Water Flux and Water Recovery 

**Figure 3:**
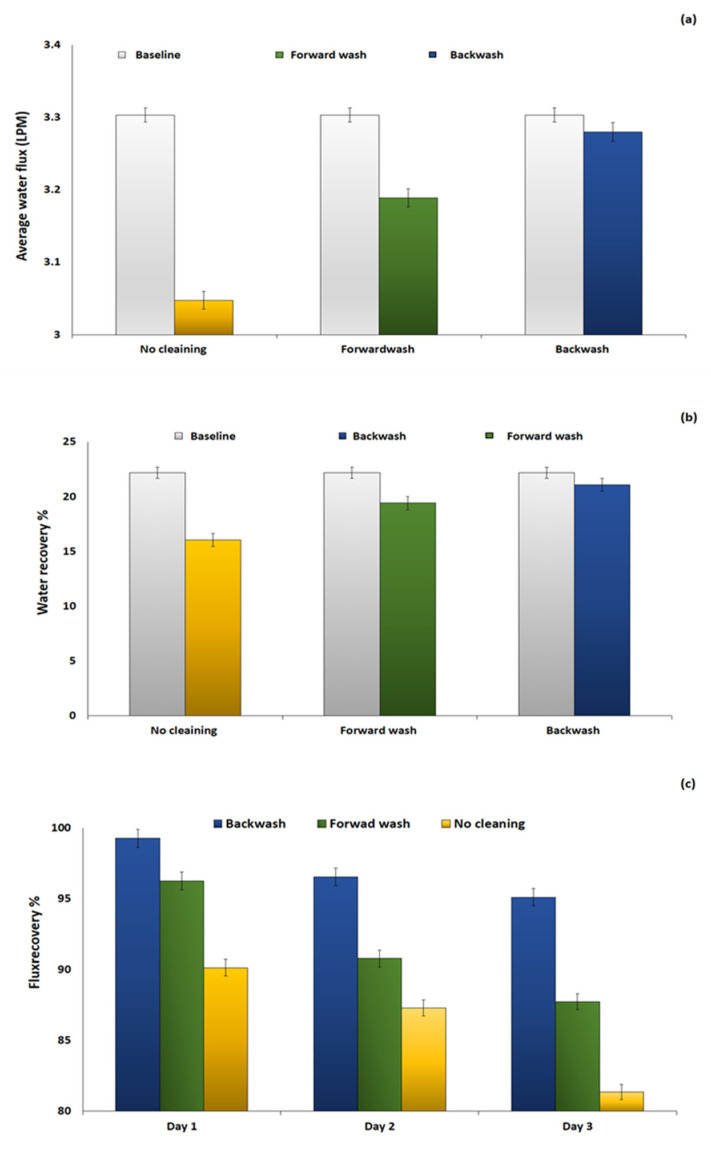
(**a**) Average water flux, (**b**) water recovery % and (**c**) flux recovery% after three consecutive FO runs. Conditions: FS = SWW, MCFDS (300 g/L) and FS and DS flowrate = 1.6 LPM at room temperature for 24 h operating time. Baseline represents the initial fertilizer-drawn forward osmosis (FDFO) experiment using a new cellulose triacetate (CTA) FO membrane, SWW as FS and MCFDS (300 g/L) at 1.6 LPM for 24 h.

The membrane fouling behavior was further investigated through the accelerated fouling experiment, using the SWW as FS and MCFDS without a cleaning protocol, each run lasting for 24 h. Then, the effect of physical cleaning on foul membrane was studied. Several studies claimed that the fouling of membranes in the FO and consequently the FDFO process is entirely reversible by physical cleaning methods. Figure 3a–c is used to depict the WF, percentage of water recovery and flux recovery percentage for forward wash (hydraulic/in situ flushing) and osmotic backwash at fixed operating conditions. The general trend shows that the generated WF after applying physical cleaning was closer to the initial WF baseline and higher than that without cleaning. This indicates that both mechanical cleaning methods were able to reduce the foul layer on the membrane surface and maintain an average WF behavior close to the baseline. However, higher WF was recovered after backwash a cleaning protocol compared to in-situ flushing. This can be attributed to the effect of reverse water movement that tends to retrieve ultra-pure water from the draw side to the feed side (saline) which tends to remove the foul layer. However, in the in situ flushing the pure water was circulated to clean the system without changing the direction of the flow. Figure 3b shows that the water recovery percentage behavior was in line with the WF trend after cleaning. Initially, the system was able to recover 22.2% of water from the SWW. Following this, the % of water recovery decreased slightly after applying physical cleaning. The backwash cleaning was able to recover 8% water from the FS, which was higher than that obtained with forward cleaning. The percentage of flux recovery was used to assess the effectiveness of backwash and forward washing by performing FO runs that lasted for three consecutive days. The system was stopped for cleaning after 24 h. 

As can be observed from Figure 3c, the decline in flux after each cycle is primarily caused by two factors. The first is the decline in the initial water flux in each cycle due to fouling. The second is the inevitable change in the physiochemical properties of the membrane surface due to residual foulants. 

Liu et al. [18] reported that the percentage of water recovery improved after applying physical cleaning in line with WF behavior [4]. Figure 3c shows that the percentage of flux recovery dropped from (99% to 95%) and (96% to 87%) when a backwash and a forward wash cleaning was implemented, respectively. This indicates that backwash cleaning is more effective in recovering the initial WF than hydraulic flushing. The higher membrane cleaning efficiency of backwashing hints that the reverse flow of water can interrupt the interaction of the foulants with the membrane layer. It has further been reported by Kim et al. [17] that backwashing can eliminate foulant–foulant and foulant–membrane adhesion with use of appropriate salts in the process. Mosta et al. [26] revealed that osmotic driving force is capable of cleaning fouled FO and recovering the permeate flux from 81% up to 98% for 1 M and 1.5 M NaCl concentration. Moreover, Lotfi et al. [13] claimed that the backwash was based on the osmotic driving force that induces water permeation across the membrane and weakened the thickness of the fouling layer on the membrane surface. Accordingly, a number of recent studies claimed that hydraulic flushing was more effective in recovering water flux and the reducing fouling layer when operating at a higher circulation flowrate [31].

Hound et al. [32] studied fouling characteristics of CTA FO membrane by using microalgae nutrients as FS, with respect to a different FO membrane orientation of AL facing the FS (AL–FS) and AL facing the DS (AL–DS). They concluded that the AL–DS mode exhibited a rapid decline in WF and a lower flux recovery after applying the physical cleaning compared to the AL–FS mode, because of internal pore clogging and inner membrane fouling, along with the ICP within the PL. Similarly, Mi et al. [10] reported that AL–FS mode showed a better WF recovery of 10% than that obtained by the AL–DS mode after physical cleaning. This gives an indication that the membrane AL has a significant influence in determining the effectiveness of physical cleaning as it may eliminate the need for chemical cleaning. 

The type of FS has a major influence in determining the required cleaning approach for the FDFO system. Studies on the FDFO system have examined the process performance by using a very contaminated FS such as a sewage water and primary wastewater [23,33]. For such system, the hydraulic flushing was insufficient to recover water flux and they suggested the need to apply a pre-treatment to the FS. Other studies on the FDFO system used brackish water as FS, reporting that backwash cleaning is an effective method compared with forward washing, and it requires minimal energy consumption, since it depends on the osmotic difference effect between the FS and DS [22]. 

The effectiveness of physical cleaning on the performance of the FDFO hints towards the reversible nature of the foulants deposited on the membrane. The absence of hydraulic pressure in the FDFO process causes a sparse and thick deposit of organic fouling on the FO membrane, which, as observed by the effectiveness of the cleaning methods, is removed by generating shear stress. Xie et al. [27] reported that gypsum scaling in the FO process is nearly recoverable, where more than 96% recovery of WF was achieved after applying in situ flushing. In addition, they found that gypsum scaling is more difficult to remove from polyamide (PA) membrane than that of cellulose acetate (CA). This indicates that the physical cleaning of the CTA FO membrane from gypsum scaling is a cost effective method, as it requires the minimum energy consumption necessary for water circulation flowrate (e.g., hydraulic flushing). However, scalable FO setup is expected to demand more energy requirement. Therefore, the power consumption by the FO process operation and physical cleaning can be further minimized by adapting a renewable source of energy power, such as the microbial desalination cell (MDC) and microbial fuel cell (MFC) [34].

#### 3.2.2. Salt Rejection 

**Figure 4:**
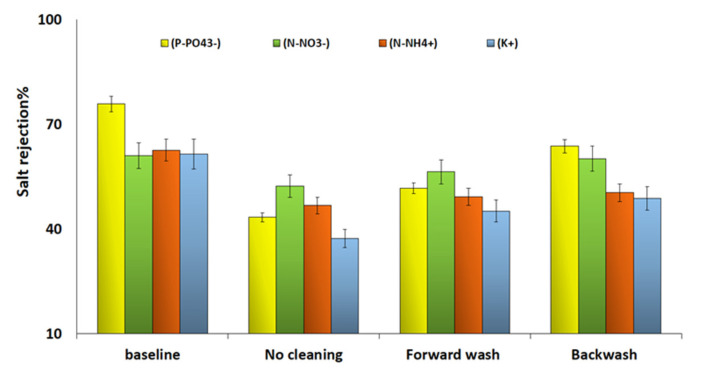
Salt rejection % of P–PO_4_^3−^, N–NO_3_^−^, N–NH_4_ and K^+^ after three consecutive FO runs. Conditions: FS = SWW, DS = MCFDS (300 g/L) and FS and DS flowrate = 1.6 LPM at room temperature for 24 h operating time. Baseline represents the initial FDFO experiment using a new CTA FO membrane, SWW as FS and MCFDS (300g/L) at 1.6LPM for 24 h.

The percentage of salt rejected (%R) of macronutrients (NH_4_^+^, NO_3_^−^, PO_4_^3−^ and K^+^) from the FS before and after using a different physical cleaning protocol were analyzed and the results are shown in Figure 4. For the MCFDS of 300 g/L, the initial %R for the different salts was found to be lower than 80% with phosphate exhibiting the highest rejection with 75%, while nitrate (NO_3_^−^) showed the lowest with 60%. A sharp decline in R% was observed for all the salt after three consecutive FO and without applying physical cleaning. After applying backwash and forward wash, results with a slight improvement in R% were observed for all salts. The general trend of R% for PO_4_^3−^ and K^+^, showed that backwash maintained the percentage salt rejection of those solutes closer to the baseline. However, the R% of (NO_3_^−^) and ammonia (NH_4_^+^) after physical cleaning showed insignificant changes.

Despite the high WF exhibited by the FDFO system, owing to the effective osmotic driving force generated by the FS (SWW) and MCFDS, however the R% of all salts ranged from 34% to 75%. The factor dictating the decline in salt rejection might be related to the type and characteristics of the membrane in term of selectivity and permeability. For instance, Jin et al. [35] reported that the CTA FO membrane showed a varying salt rejection ranging from 65% up to 95%. Other factors which may affect salt rejection is the type and the concentration of FS. Such reason can be related to this study, where the FS (SWW) is very saline and composed of macronutrient and micronutrients (Mg^2+^, Ca^2+^ and SO_4_^2−^) and therefore low salt rejection was observed. Similarly, it has been reported by McCutcheon et al. [28] that increasing the concentration of NaCl solution used as FS at fixed operating conditions reduced the salt rejection by 4%. The bench-scale FDFO system with flat sheet membrane was able to reject up to 75% of macronutrients. For practical implementation, the performance of the FDFO system in terms of R% requires considerable modification in terms of system scalability and operating conditions. 

### 3.3. Morphologies of CTA Membrane 

#### 3.3.1. SEM

**Figure 5:**
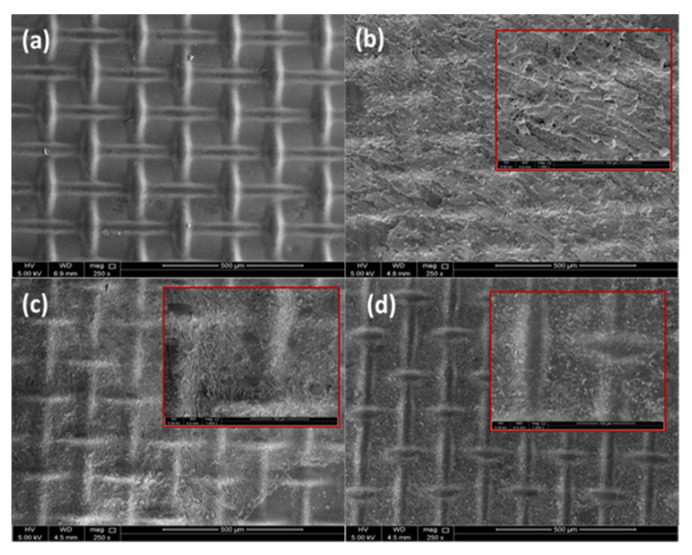
Cross-sectional SEM image of the active layer surface of the CTA FO membrane after three consecutive FO runs: l (**a**) origin; (**b**) without physical cleaning; (**c**) in situ flushing; and (**d**) osmotic backwash. Conditions: FS = SWW, DS = MCFDS (300 g/L) and FS and DS flowrate = 1.6 LPM at room temperature for 24 h operating time.

Scanning electron microscopy (SEM) was conducted to verify the results obtained thus far and in particular with regards to the fouling experiment. The SEM of the active layer (AL) surface of the CTA FO membrane was performed before and after applying physical cleaning. The results of the SEM are shown in Figure 5a–d. The microscopic measurement showed a thick fouling layer formed on the AL surface of the membrane when no cleaning protocol was performed, as seen in Figure 5b. This explains the drop in WF from 3.3 LMH to 2.68 LMH after three days of operation, whereas a reduction of the fouling layer formation was observed after applying a cleaning protocol, as shown in Figure 5c,d. This indicates that the FO membrane surface could be physically cleaned or reduced from the foul deposited on the membrane surface. Moreover, negligible inorganic fouling can be observed on the AL surface after several runs along with the use of osmotic backwash after each run. In contrast, Figure 5c shows that there is a deposit inorganic foul on the AL surface even after applying forward wash (hydraulic flushing). This verifies our previous findings about the increased efficiency of osmotic backwashing over hydraulic flushing. The same findings were reported by Suwaileh et al. [31] suggesting the use of a backwash cleaning protocol at low circulation flow to overcome fouling when performing FDFO desalination for brackish water. Liu et al. [18] observed through SEM, a crystal layer deposit on the membrane surface after applying hydraulic flushing using DI water for both FS and DS after treating radioactive wastewater. Accordingly, they suggested applying hydraulic flushing combined with other cleaning methods. This implies that the type of cleaning technique depends on the type of FS. 

#### 3.3.2. AFM

**Figure 6:**
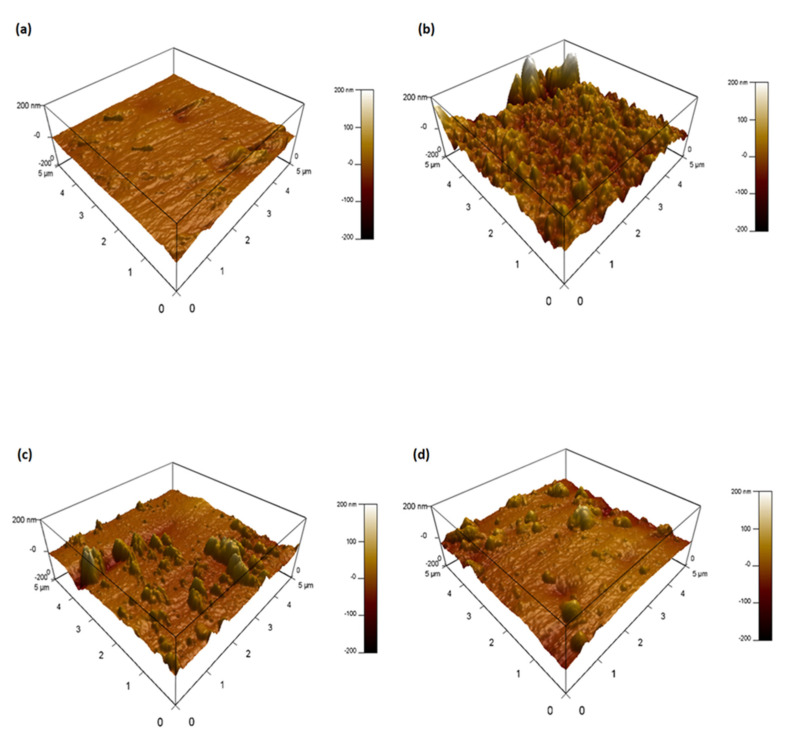
Atomic force microscopy (AFM) image of the active layer surface of CTA FO membrane after three consecutive FO runs: l (**a**) origin; (**b**) without physical cleaning; (**c**) in situ flushing; and (**d**) osmotic backwash. Conditions: FS = SWW, DS = MCFDS (300 g/L) and FS and DS flowrate = 1.6 LPM at room temperature for 24 h operating time.

Atomic force microscopy (AFM) was performed to support the results obtained through SEM. The results are depicted in Figure 6a–d and present the surface roughness of the AL of the CTA FO membrane before and after physical cleaning. The height distribution of the AFM images represents the root mean square (RMS) (*R_q_*) that we consider being the most used parameter to evaluate for surface roughness and the standard *R_q_* value corresponds to 7 nm. The AFM image reveals that the *R_q_* value of the origin AL of CTA FO membrane was 7.78 nm, whereas the *R_q_* values of no clean, backwash and forward wash surfaces were 42.22 nm, 16.629 nm and 18.088 nm, respectively. The foul surface exhibited the highest *R_q_* value, due to the thick foul layer observed on the membrane surface in Figure 6b. This further verifies the decline in flux following 3 days of operation. The AFM images obtained after the physical cleaning of the membranes showed a significant reduction in the foulants adhering to the membrane. However, as shown in Figure 6c, noticeable organic fouling remains on the membrane after in situ flushing. In contrast, Figure 6d depicting the AFM imaging after osmotic backwash showed minimal fouling and corresponded to the lowest *R_q_* value. This concludes that osmotic backwash was strikingly effective in disrupting foulant–membrane interaction to minimize FO fouling. Membrane roughness caused by foulants, were reported to have a higher fouling rate due to the increased adhesion tendency of the foulants. However, osmotic backwashing is concluded to be an effective method to curb the excessive deposition of foulant. 

#### 3.3.3. FTIR

**Figure 7:**
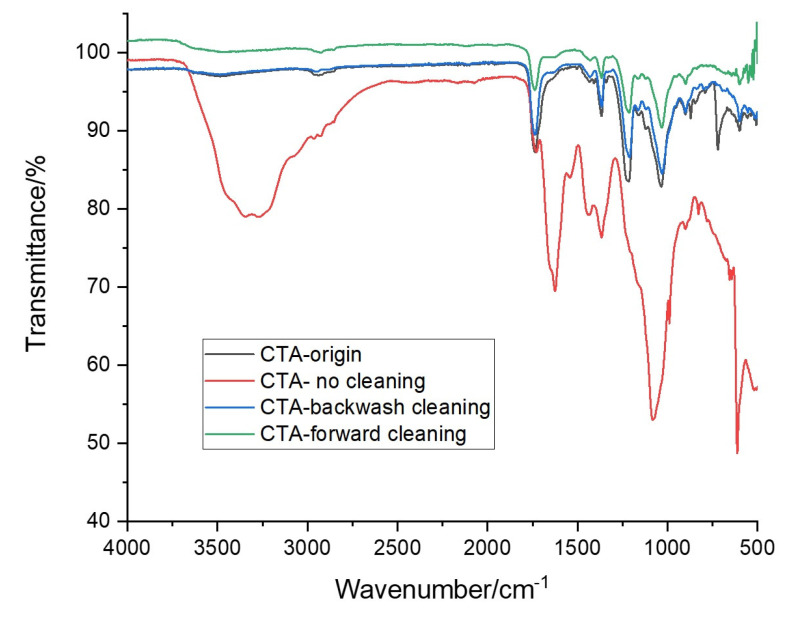
FTIR spectra of the active layer surface of the CTA FO membrane after three consecutive FO runs. Conditions: FS = SWW, DS = MCFDS (300 g/L) and FS and DS flowrate = 1.6 LPM at room temperature for 24 h operating time.

Fourier Transform infrared (FTIR) was performed to characterize the chemical properties of the foul layer. Figure 7 shows the changes in the FTIR spectra of the CTA membrane surface functional group due to the fouled layer. The FTIR spectra of the origin CTA membrane surface confirmed the presence of (C–O) stretching in the hydroxyl functional group at a wavenumber of 1367.49 cm^−1^ and ester functional group (C–O) stretching in cellulose triacetate at a wavenumber of 1735.86 cm^−1^, as identified in the literature [8]. After three consecutive FO runs without cleaning, a significant change was observed in the FTIR spectra at wavenumbers of 3226.51 cm^−1^, 1735.86 cm^−1^, 1367.49 cm^−1^ and 1212.49 cm^−1^. This indicate the presence of a thick foul layer deposited on the surface of the CTA membrane and after applying physical cleaning, the FTIR spectra were shifted slightly upward compared with FTIR curve of the origin CTA membrane surface. In accordance with the above results, the FTIR curve of the backwash showed a minor deviation from the origin CTA membrane surface. This proves that the interaction occurred between the fouling layer and the AL of the CTA membrane surface. Moreover, it demonstrates that osmotic backwash is an effective method to minimize the foul layer and created by high slain FS and able to recover the initial WF generated by the FDFO system. The same finding was reported by Mi et al. [10] and Xie et al. [27] that the gypsum scaling coverage on the CTA membrane caused no interaction with the CTA membrane surface by the mainly unchanged ratio of wavenumbers after applying physical cleaning.

## 4. Conclusions

The undeniable advantage of FDFO should not be hindered by the inevitable membrane fouling experienced by the process. The present study evaluates the impact of membrane fouling on the performance of the FDFO process. The type of feed solution used had a significant impact on the fouling of the membrane. Foulant buildup due to concentration polarization impacted the effective osmotic driving force and subsequently the water flux. The continuous dilution of recovered water from the feed side to the draw side further contributed to the drop in flux when no cleaning protocol was performed. The lower SWF of SWW was a result of the lower osmotic pressure difference between the FS and the DS as compared to deionized water thus resulting in lower flux. Osmotic backwashing exhibited a better cleaning efficiency of the membrane fouling compared against in situ flushing; both methods, however, provided a satisfactory water recovery percentage. The salt rejection was found to be below 80%, mainly attributed to the decline in water flux without any decline in salt flux. Lastly, the SEM, AFM imaging and FTIR spectra conducted verified the attained results.

## Figures and Tables

**Table 1:** Synthetic wastewater (SWW) characteristics.

Composition	Units	SWW
pH		7.4
EC (Electric Conductivity)	mS/cm	60.33
π	bar	37.96
TDS (Total Dissolved Solids)	mg/L	54,297
TOC (Total Organic Content)	mg/L	1333
COD (Chemical Oxygen Demand)	mg/L	861.66
Ammonia (NH_3_)	mg/L	1370.3
Nitrate (NO_3_^−^)	mg/L	2100.65
Phosphate (PO_4_^3−^)	mg/L	3125
Potassium (K)	mg/L	4081.15
Sulfate SO_4_^2−^	mg/L	1364.85
Chloride (Cl)	mg/L	20,550.75
Sodium (Na)	mg/L	12,376.25
Calcium (Ca)	mg/L	265.4
Magnesium (Mg)	mg/L	179.5

**Table 2:** Composition of commercial fertilizer.

Fertilizer	Weight Percent%
Total Nitrogen (N)	20
Ammonia (NH_4_^+^-N)	3.9
Nitrate (NO_3_^−^-N)	3.6
Urea-N	12.6
Phosphorus peroxide (P_2_O_5_-P)	20
Potassium oxide (K_2_O-K)	20

## Abbreviations

Active layer	AL
Atomic Force Microscopy	AFM
Cellulose triacetate	CTA
Concentration polarization	CP
Concentrative external concentration polarization	CECP
Deionized water	DI water
Dilutive internal concentration polarization	DICP
Draw solution/solute	DS
External concentration polarization	ECP
Feed solution	FS
Fertilizer draw solution/solute	FDS
Forward osmosis	FO
Fourier Transform infrared spectroscopy	FTIR
Internal concentration polarization	ICP
Multi- components fertilizer	MCF
Multi- components fertilizer draw solution	MCFDS
Porous layer	PL
Root mean square	RMS or Rq
Salt rejection percentage	R%
Scanning Electron Microscopy	SEM
Synthetic wastewater	SWW
Water flux	WF
